# Molecular Insights Into the Evolutionary Pathway of *Vibrio cholerae* O1 Atypical El Tor Variants

**DOI:** 10.1371/journal.ppat.1004384

**Published:** 2014-09-18

**Authors:** Eun Jin Kim, Dokyung Lee, Se Hoon Moon, Chan Hee Lee, Sang Jun Kim, Jae Hyun Lee, Jae Ouk Kim, Manki Song, Bhabatosh Das, John D. Clemens, Jean William Pape, G. Balakrish Nair, Dong Wook Kim

**Affiliations:** 1 Department of Pharmacy, College of Pharmacy, Hanyang University, Ansan, Korea; 2 Institute of Pharmacological Research, Hanyang University, Ansan, Korea; 3 Laboratory Science Division, International Vaccine Institute, Seoul, Korea; 4 Translational Health Science and Technology Institute, Gurgaon, Haryana, India; 5 International Centre for Diarrhoeal Disease Research, Dhaka, Bangladesh; 6 UCLA Fielding School of Public Health, Los Angeles, California, United States of America; 7 Division of Infectious Diseases, Department of Medicine, Weill Cornell Medical College, New York, New York, United States of America; 8 Les Centres GHESKIO, Port-au-Prince, Haïti; Harvard University, United States of America

## Abstract

Pandemic *V. cholerae* strains in the O1 serogroup have 2 biotypes: classical and El Tor. The classical biotype strains of the sixth pandemic, which encode the classical type cholera toxin (CT), have been replaced by El Tor biotype strains of the seventh pandemic. The prototype El Tor strains that produce biotype-specific cholera toxin are being replaced by atypical El Tor variants that harbor classical cholera toxin. Atypical El Tor strains are categorized into 2 groups, Wave 2 and Wave 3 strains, based on genomic variations and the CTX phage that they harbor. Whole-genome analysis of *V. cholerae* strains in the seventh cholera pandemic has demonstrated gradual changes in the genome of prototype and atypical El Tor strains, indicating that atypical strains arose from the prototype strains by replacing the CTX phages. We examined the molecular mechanisms that effected the emergence of El Tor strains with classical cholera toxin-carrying phage. We isolated an intermediary *V. cholerae* strain that carried two different CTX phages that encode El Tor and classical cholera toxin, respectively. We show here that the intermediary strain can be converted into various Wave 2 strains and can act as the source of the novel mosaic CTX phages. These results imply that the Wave 2 and Wave 3 strains may have been generated from such intermediary strains in nature. Prototype El Tor strains can become Wave 3 strains by excision of CTX-1 and re-equipping with the new CTX phages. Our data suggest that inter-chromosomal recombination between 2 types of CTX phages is possible when a host bacterial cell is infected by multiple CTX phages. Our study also provides molecular insights into population changes in *V. cholerae* in the absence of significant changes to the genome but by replacement of the CTX prophage that they harbor.

## Author Summary

In this report, we suggest a genetic mechanism of how the *V. cholerae* atypical El Tor variants were generated from classical and prototype El Tor biotype strains. An intermediary strain, containing the CTX-1 and CTX-2 prophages, was identified among the clinical isolates that were collected in 1991, when the atypical strains emerged. This strain can be converted into various Wave 2 atypical El Tor strains by eliminating prototype components, CTX-1 and RS1. Further, new types of the CTX phage genome can be generated from the intermediary strain by inter-chromosomal recombination between CTX phages and recombination between the CTX phage and RS1. These new CTX phages can be transduced into other El Tor strains, transforming them into Wave 3 atypical strains. This is a demonstrated instance of how a single-segment-genome CTX phage re-organizes its genome through recombination between different types of phage, leading to generation of new phage variants and atypical El Tor strains.

## Introduction

*Vibrio cholerae* O1 serogroup strains have been categorized into 2 biotypes - classical and El Tor -based on microbiological properties and the CTX prophage that they harbor [1], [2]. Classical biotype strains contain the classical CTX prophage (CTX^cla^), and El Tor strains are believed to contain the El Tor CTX prophage (CTX^El Tor^, or CTX-1).

The CTX phage comprises 10 genes (*rstR*, *rstA*, *rstB*, *psh*, *cep*, *orfU*, *ace*, *zot*, *ctxA*, and *ctxB*). Whereas *rstR* is phage type-specific, other genes differ between phages by several SNPs, except for *ctxA*, which is identical in the 2 phages [3]. The binding subunit of cholera toxin (CTB) is encoded by *ctxB*, and the CTBs between phages differ by 2 of their 125 amino acids (residues 39 and 68) [1]. An evolutionary model of pathogenic *V. cholerae* O1 strains, in which El Tor biotype strains acquired only the El Tor CTX phage, whereas the classical strains obtained the classical phage, has been widely accepted [4].

Atypical El Tor variants, defined as El Tor biotype strains that produce classical cholera toxin, were first recognized in 2006, and several atypical El Tor variants have since been reported [5]–[7]. Two atypical CTX phages that contain *ctxB* ^cla^ have been reported among atypical El Tor strains [8], the first of which was discovered in *V. cholerae* clinical isolates of cholera outbreaks in Mozambique in 2004 and was later found to have existed in South Asian countries since the early 1990s [9]. This CTX phage contains the classical biotype-specific *rstR* (*rstR* ^cla^) and *ctxB* (*ctxB* ^cla^), and was thus considered CTX^cla^. However, it was later found to contain other genes of CTX-1 and was renamed CTX-2 [10]. *V. cholerae* strains that contain CTX-2 harbor a tandem repeat of CTX-2 on chromosome 2. Based on the analysis of SNPs in the genome and the CTX phage they harbor, the El Tor strains have been categorized into 3 Waves [10]. The prototype El Tor strains that contain CTX-1 are considered Wave 1 strains. The strains contain CTX-2 constitute a phylogenetic subgroup among the seventh cholera pandemic strains by genome analysis and are therefore categorized as Wave 2 strains [10].

**Figure 1:**
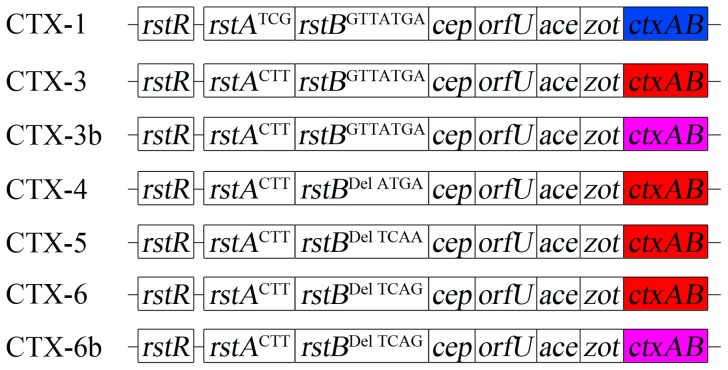
Genetic map of CTX phages of *Vibrio cholerae* O1 El Tor Wave 3 atypical strains aligned with Wave 1 strain N16961 (genomes not shown to scale). *ctxAB* of N16961 (*ctxB* genotype 3) is shown in blue, and *ctxAB*s (classical *ctxB*, or *ctxB* genotype 1) of CTX-3, CTX-4, CTX-5, and CTX-6 are shown in red. *ctxB* of genotype 7 (Haiti strain type) of CTX-3b and CTX-6b is shown in purple. SNPs of *rstA* and *rstB* are shown as superscripts (position 927, 933, and 942 of *rstA* and positions 74–76, 87, 93, 105, and 189 of *rstB*). Del indicates 3-nucleotide (74–76 of *rstB*) deletion.

The second atypical CTX phage (CTX-3) was first reported in Vietnam in 2007 and has the same genetic structure and sequence as CTX-1, with the exception of SNPs in *rstA* and *ctxB* (Figure 1) [11]. Strains that harbor CTX-3 typically contain a satellite phage, RS1, followed by a CTX-3 on chromosome 1 [6]. These strains have existed since the early 1990s on the Indian subcontinent [6]. A variant of CTX-3 that contains a new type of *ctxB* (*ctxB* genotype 7) emerged from India in 2006 (Figure 1). In addition to 2 SNPs in *ctxB* ^cla^ of CTX-3 compared with *ctxB* ^El Tor^ of CTX-1, CTX-3b has an additional SNP (nucleotide position 58) at amino acid residue 20 [12]. Most current global clinical isolates of *V. cholerae* are atypical El Tor variants that harbor CTX-3 or CTX-3b [6]. A recent surveillance study in India has shown that strains containing CTX-3b have been gradually replacing strains with CTX-3, as isolates that contain CTX-3b constitute 93.3% of all isolates that were collected in 2011 [13]. *V. cholerae* strains that contain CTX-3 or CTX-3b are distinguished from Wave 2 strains, based on the CTX phage and genomic variations, and are thus categorized as Wave 3 strains of the seventh cholera pandemic [10].

The catastrophic cholera outbreak in Haiti in 2010 was caused by a Wave 3 strain that contained *ctxB* genotype 7 [10], [14], [15]. We noted the CTX phage of Haitian strains differed from CTX-3b by several SNPs. We surveyed Wave 3 *V. cholerae* strains that were collected in Kolkata, India from 2003–2007 to verify that the Haitian-type CTX phage had existed earlier. We identified a CTX phage that contains identical SNPs as the Haitian-type CTX phage (except for *ctxB*) and other variant CTX phages. The SNPs of these variant CTX phages appeared to originate from the RS1 satellite phage, implying that the variant CTX phages were generated by recombination of homologous genes (*rstR*, *rstA*, and *rstB*) between RS1 and CTX.

V212-1, a strain that bears CTX-1 and CTX-2, was collected when atypical strains first appeared (Table 1) and was considered to be an intermediary between prototype and atypical El Tor strains [16]. In this study, we demonstrate that Wave 2 strains can be generated directly from V212-1 by progressive elimination of CTX-1 and RS1 through intra-strand recombination. Further, novel mosaic CTX phages can be produced by an inter-chromosomal recombination between 2 different CTX prophages in V212-1, and the mosaic phages can be transmitted to new host bacteria, converting them into Wave 3 strains. These results indicate that the intra-strand and inter-strand recombination between CTXs and RS1 element played an important role in the evolution of CTX phage and *V. cholerae*, as previously suggested [17].

**Table 1:** Genetic variations in RS1 and CTX prophages aligned with CTX-1 of N16961.

CTX type	Strain name	Origin	*rstA*													*rstB*														ToxR repeat	*ctxB*			Sequence information
			27	162	183	258	345	516	540	579	609	774	927	933	942	74–76	87	93	105	189	285	288	341	360	364	366–368	371–372	379	*381*		58	115	203	
RS1	N16961	Bangladesh, 1975	**·**	**·**	**·**	**·**	**·**	**·**	**·**	**·**	**·**	**·**	C	T	T	Δ	T	C	A	G	**·**	**·**	**·**	G	A	Δ	Δ	A	Δ					AE003852
	V212-1	India, 1991	**·**	**·**	**·**	**·**	**·**	**·**	**·**	**·**	**·**	**·**	C	T	T	Δ	T	C	A	G	**·**	**·**	**·**	G	A	Δ	Δ	A	Δ					KF664566
CTX-1	N16961	Bangladesh, 1975	C	C	C	G	G	G	A	T	T	C	T	C	G	GTA	A	T	G	A	A	C	G	A	C	ACC	TT	T	A	4	C	T	T	AE003852
	V212-1	India, 1991	**·**	**·**	**·**	**·**	**·**	**·**	**·**	**·**	**·**	**·**	**·**	**·**	**·**	**···**	**·**	**·**	**·**	**·**	**·**	**·**	**·**	**·**	**·**	**···**	**··**	**·**	***·***	4	**·**	**·**	**·**	KF664566
CTX-2	B33	Mozambique, 2004	T	T	A	C	**·**	**·**	**·**	**·**	**·**	**·**	**·**	**·**	**·**	**···**	**·**	**·**	**·**	**·**	**·**	**·**	T	**·**	**·**	**···**	**··**	**·**	***·***	4	**·**	C	C	GQ485644
	V212-1	India, 1991	T	T	A	C	**·**	**·**	**·**	**·**	**·**	**·**	**·**	**·**	**·**	**···**	**·**	**·**	**·**	**·**	**·**	**·**	**·**	**·**	**·**	**···**	**··**	**·**	***·***	4	**·**	C	C	KF664567
CTX-3	IB4122	Vietnam, 2007	**·**	**·**	**·**	**·**	**·**	**·**	**·**	**·**	**·**	**·**	C	T	T	**···**	**·**	**·**	**·**	**·**	**·**	**·**	**·**	**·**	**·**	**···**	**··**	**·**	***·***	4	**·**	C	C	GQ485652
	IB4322	India, 2004	**·**	**·**	**·**	**·**	**·**	**·**	**·**	**·**	**·**	**·**	C	T	T	**···**	**·**	**·**	**·**	**·**	**·**	**·**	**·**	**·**	**·**	**···**	**··**	**·**	***·***	5	**·**	C	C	GQ485650
	IB4768	Djibouti, 2007	**·**	**·**	**·**	**·**	**·**	**·**	**·**	**·**	**·**	**·**	C	T	T	**···**	**·**	**·**	**·**	**·**	**·**	**·**	**·**	**·**	**·**	**···**	**··**	**·**	***·***	3	**·**	C	C	ERS013205
	IB4710	India, 2004	**·**	**·**	**·**	**·**	**·**	**·**	**·**	**·**	**·**	**·**	C	T	T	**···**	**·**	**·**	**·**	**·**	**·**	**·**	**·**	**·**	**·**	**···**	**··**	**·**	***·***	4	**·**	C	C	KJ540257
CTX-3b	IB4642	India, 2006	**·**	**·**	**·**	**·**	**·**	**·**	**·**	**·**	**·**	**·**	C	T	T	**···**	**·**	**·**	**·**	**·**	**·**	**·**	**·**	**·**	**·**	**···**	**··**	**·**	***·***	4	A	C	C	GQ485651
	IB4712	India, 2009	**·**	**·**	**·**	**·**	**·**	**·**	**·**	**·**	**·**	**·**	C	T	T	**···**	**·**	**·**	**·**	**·**	**·**	**·**	**·**	**·**	**·**	**···**	**··**	**·**	***·***	4	A	C	C	KJ540258
CTX-4	IB4563	India, 2007	**·**	**·**	**·**	**·**	**·**	**·**	**·**	**·**	**·**	**·**	C	T	T	Δ	**·**	**·**	**·**	**·**	**·**	**·**	**·**	**·**	**·**	**···**	**··**	**·**	***·***	4	**·**	C	C	KJ540260
CTX-5	IB4247	India, 2003	**·**	**·**	**·**	**·**	**·**	**·**	**·**	**·**	**·**	**·**	C	T	T	Δ	T	C	A	**·**	**·**	**·**	**·**	**·**	**·**	**···**	**··**	**·**	***·***	3	**·**	C	C	KJ540261
	IB4405	India, 2005	**·**	**·**	**·**	**·**	**·**	**·**	**·**	**·**	**·**	**·**	C	T	T	Δ	T	C	A	**·**	**·**	**·**	**·**	**·**	**·**	**···**	**··**	**·**	***·***	4	**·**	C	C	KJ540262
CTX-6	IB4540	India, 2007	**·**	**·**	**·**	**·**	**·**	**·**	**·**	**·**	**·**	**·**	C	T	T	Δ	T	C	A	G	**·**	**·**	**·**	**·**	**·**	**···**	**··**	**·**	***·***	4	**·**	C	C	KJ540263
CTX-6b	IB5230	Haiti, 2010	**·**	**·**	**·**	**·**	**·**	**·**	**·**	**·**	**·**	**·**	C	T	T	Δ	T	C	A	G	**·**	**·**	**·**	**·**	**·**	**···**	**··**	**·**	***·***	5	A	C	C	KJ540264
CTX^cla^	O395	India, 1965	T	T	A	C	T	A	G	C	C	T	**·**	**·**	**·**	Δ	T	C	G	A	G	T	**·**	**·**	**·**	**···**	**··**	**·**	***·***	7	**·**	C	C	CP000626

ToxR repeat sequence is TTTTGAT.

SNPs of *rstA*, *rstB* and *ctxB* are indicated. The number of ToxR binding repeats between *zot* and *ctxA* is also shown. Dots indicate identical sequences as CTX-1, and Δ indicates deletion of nucleotide(s).

## Results

### Variants of mosaic CTX phages in *V. cholerae* O1 El Tor Wave 3 atypical strains

The genome sequences of CTX prophages indicated that the CTX phage genome can be classified into several types, based on SNPs of *rstA*, *rstB*, and *ctxB*, as suggested by Mutreja *et al.* [10]. The CTX-3 prophages in *V. cholerae* isolates belonging to Wave 3 strains that were collected in Vietnam and India contained 3 SNPs (nucleotides 927, 933, and 942) in *rstA* compared with CTX-1 of N16961 and V212-1, as shown in Table 1 [8], [18]. The CTX prophage in Haitian isolates that were collected during the 2010 cholera outbreak was believed to be CTX-3b, which contains *ctxB* genotype 7. However, SNPs (3-nucleotide [GTA] deletion of positions 74–76 and SNPs at positions 87, 93, 105, and 189) in *rstB*, in addition to the 3 SNPs of *rstA*, were identified by sequencing the CTX genome of Haitian strains (Table 1). The difference in SNPs between CTX-3 and Haitian-type CTX suggests that more CTX variants exist among Wave 3 *V. cholerae* strains and that the CTX phages in Wave 3 strains can be classified further.

Because the GTA deletion in *rstB* could be used as a marker for identifying the Haitian-type CTX phage genome, a PCR strategy was designed to detect the presence and absence of this deletion (Figure S1). *rstB* PCR and DMAMA-PCR for *ctxB* were performed on 365 clinical isolates that were collected in Kolkata, India between 2003 and 2007 [13], [19]. Most clinical isolates contained CTX-3 or CTX-3b, whereas 11 had GTA-deleted CTX prophage (Table S1).

CTX-3 was the most prevalent type; CTX-3b emerged in 2006, as previously reported [12]; and CTX-4, CTX-5, and CTX-6 were identified in 2004, 2003–2005, and 2007, respectively. The 20 clinical isolates from the cholera outbreak in Haiti in 2010 contained the same CTX prophage that could be categorized as CTX-6b, according to this scheme. However, strains that contained CTX-6b had not been identified in India by 2007.

Notably, the location of the SNPs in *rstA* and *rstB* in these CTX phages appear to be of RS1, implying that the CTX phage genomes are mosaics of CTX-1 and RS1 (Table 1).

### V212-1, an intermediary strain between prototype and atypical El Tor strains

DNA sequence analysis of CTX-2 in Wave 2 strains and various CTX phages in Wave 3 suggested that these phages in atypical *V. cholerae* El Tor strains are mosaics of CTX-1, CTX^cla^, and RS1 (Table 1) [18], [20]. It is unlikely that genetic exchange occurred between viral genomes independently of a host bacterial cell. Thus, we examined whether an intermediate strain that contained various CTX types was the origin of atypical strains. The infection of a classical strain with CTX-1, resulting in a single strain that harbors both CTX types is possible *in vitro* but, no clinical isolates or reference strains that contained both types of CTX in a single bacterial cell are available [17], [21]_ENREF_15. Instead, an intermediary strain, V212-1, that contains CTX-1 and CTX-2 has been reported [16].

We determined and verified the exact array of CTX and RS1 in V212-1—TLC:RS1:CTX-1:RS1 on chromosome 1 and CTX-2:CTX-2 on chromosome 2—by sequencing (Table 2). CTX-1 on chromosome 1 was identified as an authentic CTX-1, as the SNPs in *rstA* and *rstB* were identical to those of CTX-1 in N16961.

**Table 2:** Genetic information on *V. cholerae* strains and pCTXs generated in this study.

Strains and plasmids	Genetic structure			
	Chromosome 1		Chromosome 2	
	CTX array	GenBank accession	CTX array	GenBank accession
V212-1 derivatives				
V212-1	TLC:RS1:CTX-1:RS1	KF664566	CTX-2:CTX-2	KF664567
PM1	TLC:CTX-1:RS1	KF664568	CTX-2:CTX-2	
PM2	TLC:RS1:RS1	KF664569	CTX-2:CTX-2	
PM3	TLC:RS1	KF471410	CTX-2:CTX-2	
PM4	TLC	KF664570	CTX-2:CTX-2	
PM5	No TLC, No element	KF664571	CTX-2:CTX-2	
PM6	TLC:RS1:CTX-1:RS1		CTX-2	KF664572
PM7	TLC:RS1:CTX-1kan:RS1	KF664573	CTX-2:CTX-2	
PM8	TLC:CTX-1kan:RS1	KF664574	CTX-2:CTX-2	
PM9	TLC:RS1:CTX-1:RS1		CTX-2kan:CTX-2	KF664575
PM10	TLC:RS1:CTX-1:RS1		CTX-2:CTX-2kan	KF664576
PM11	TLC:CTX-3^#^:RS1	KJ540266	CTX-2kan:CTX-2	
PM12	TLC:CTX-5^#^:RS1	KJ540267	CTX-2kan:CTX-2	
PM13	TLC:CTX-6^#^:RS1	KJ540268	CTX-2kan:CTX-2	
pCTX				
pCTX-1kan	pCTX-Kan generated from PM7	KF664579		
pCTX-1-1kan	pCTX-Kan generated from PM8	KF664580		
pCTX-1*kan	pCTX-Kan generated from PM9	KJ540269		
pCTX-3kan	pCTX-Kan generated from PM11	KJ540270		
pCTX-5kan	pCTX-Kan generated from PM12	KJ540271		
pCTX-6kan	pCTX-Kan generated from PM13	KJ540272		
N16961 derivatives				
PM14	TLC:RS1	KJ540273	No element	
PM15	TLC:RS1:CTX1kan	KJ540274	No element	
PM16	TLC:RS1:CTX1*kan	KJ540275	No element	
PM17	TLC:RS1:CTX3kan	KJ540276	No element	
PM18	TLC:RS1:CTX5kan	KJ540277	No element	
PM19	TLC:RS1:CTX6kan	KJ540278	No element	

### Generation of Wave 2 strains from V212-1

Whole-genome sequence analysis and the presence of a tandem repeat of CTX-2 on chromosome 2 indicate that V212-1 belongs to the Wave 2 strains [10]. However, V212-1 could be considered as an intermediary strain between Wave 1 and Wave 2 strains, because it also contained CTX-1 on chromosome 1. The generation of previously reported Wave 2 strains from V212-1 by the excision of CTX-1 and RS1 and, ultimately, excision of the entire RS1:CTX-1:RS1 array was determined as follows.

**Figure 2:**
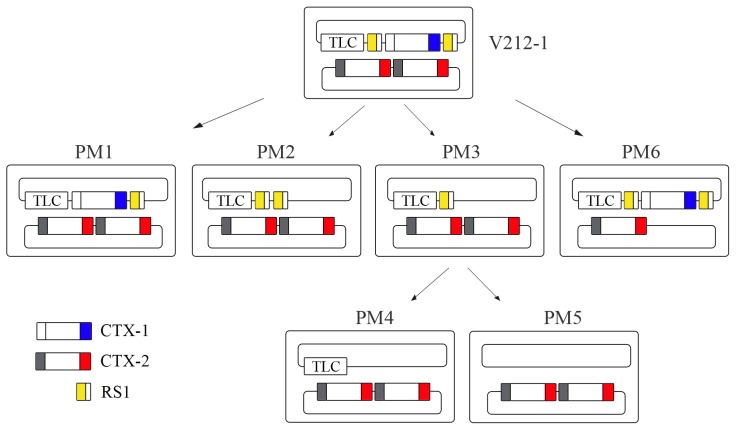
Generation of Wave 2 strains by excision of CTX-1 and RS1 from V212-1. V212-1 can be converted into several variants that belong to Wave 2 strains by excision of RS1s, CTX-1, and TLC (toxin-linked cryptic) from chromosome 1. Six variants of V212-1—PM1, PM2, PM3, PM4, PM5, and PM6—were generated in this report. Although PM4 and PM5 were generated from PM3 in this report, they could be generated directly from V212-1. RS1 is shown as a rectangle divided into 2 parts: RS2 (yellow) and *rstC* (white). CTX is shown as a rectangle divided into 3 parts: RS2 (white for CTX-1, grey for CTX-2), core, and *ctxB* (El Tor type *ctxB* is shown in blue and classical type *ctxB* is shown in red).

The removal of CTX-1 and RS1 was monitored in a recombinant strain V212-1CVD that was constructed by inserting a pCVD442-derived plasmid, pCVDrstRET, into *rstR* of CTX-1 of V212-1 (Figure S2). Three derivatives of V212-1 (PM1–PM3) were generated by excision of the first RS1, CTX-1, and CTX-1:RS1, respectively from chromosome 1 (Figure 2 and Figure S2). The overall excision rate was estimated to be 1/(4.4×10^3^), and the efficiency of generation of each array varied (Table 3). The most frequently obtained derivative was a strain that contained a solitary RS1.

**Table 3:** Frequency of generation of each array by excision of CTX-1, RS1, CTX-1:RS1 from chromosome 1 of V212-1CVD and V212-1CVD *recA* ^−^.

	RS1-CTX-RS1	RS1-RS1	CTX-RS1	RS1	Total Recombinants
V212-1CVD 2×10^5^ cells Inoculated	0	4	3	39	46
	0	10	6	37	53
	0	3	1	18	22
	0	10	7	45	62
	0	7	6	29	42
	0%	1/(2.9×10^4^)	1/(4.3×10^4^)	1/(6.0×10^3^)	1/(4.4×10^3^)
V212-1CVD *recA* ^−^ 2×10^6^ cells Inoculated	0	11	28	5	44
	0	16	17	8	41
	0	12	23	7	42
	0	9	16	5	30
	0	13	21	6	40
	0%	1/(1.6×10^5^)	1/(9.5×10^4^)	1/(3.2×10^5^)	1/(5.0×10^4^)

Results of five independent experiments are shown for each strain.

Each array can be generated by recombination events shown in Figure S2.

The excision of RS1 and/or CTX-1 from chromosome 1 seemed to be mainly by a homologous recombination, since the recombination frequency was reduced 2-fold, 5-fold, and 50-fold in *recA* ^−^ background depending on the recombination position (Table 3). However, the frequency of recombination was not reduced evenly, indicating the deletion of RS1 and/or CTX might be mediated also by other mechanism.

**Figure 3:**
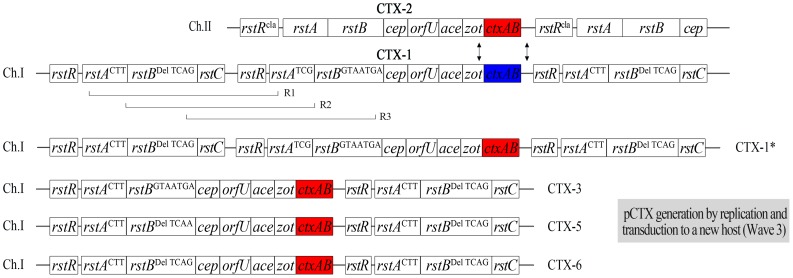
The generation of new mosaic CTX phages from V212-1 by inter-strand recombination between CTX phages and intra-strand recombination between CTX-1 and RS1 on chromosome 1. A double crossover recombination event between 2 prophages on each chromosome of V212-1 (indicated by arrows) results in the generation of the CTX-1* prophage which contains *ctxB* ^cla^ on chromosome 1. Intra-stand recombination between CTX-1* and RS1 generates a mosaic CTX prophage. Depending on the recombination position (shown as R1, R2, and R3), CTX-3, -5, and -6 can be generated (CTX-4 can also be generated, but not shown in this figure). The infectious CTX-3, -5, or -6 virions can be transduced to a new host to give rise to Wave 3 strains.

Whereas the CTX prophage of strain PM1 was identified to be the authentic CTX-1 by DNA sequencing, 3 other variant CTX phages were obtained among the derivatives that contain the CTX:RS1 array (Figure 3 and Table 2). These CTX variants can be generated by various recombination events between the RS2 region (*rstR*, *rstA*, and *rstB*) of CTX-1 and RS1 (Figure 3). The SNPs in *rstA* and *rstB* of these CTX variants were identical to the CTX-3, CTX-5, and CTX-6 prophages of clinical isolates, respectively, and were thus designated CTX-3^#^, CTX-5^#^, and CTX-6^#^ to clarify that they contained *ctxB* ^El Tor^ (Table 2). These variant strains were used later to produce pCTX-3kan, pCTX-5kan, and pCTX-6kan.

Two additional recombinant strains of V212-1, in which pCVDrstRET was inserted into *rstR* in each RS1, were constructed to examine the excision of CTX-1 and RS1 further. From a recombinant strain that contained pCVDrstRET in the first RS1, the overall excision rate of RS1 and/or CTX-1 was 1/(4.4×10^4^). Of the resulting 3 arrays (RS1:CTX-1:RS1, CTX-1:RS1, and RS1) from this strain, CTX-1:RS1 array was the most frequently produced. In a strain that contained the pCVDrstRET in the second RS1, the overall excision rate was 1/(4.7×10^5^) and the solitary RS1 array was primarily generated.

No strain that had lost the entire array of chromosome 1 was produced directly from V212-1; therefore, stepwise excision of the entire array was tested in strain PM3. pCVDrstRET was inserted into the *rstR* of RS1 in PM3 to construct PM3CVD. A strain that had lost RS1 (PM4) and 4 strains (one of them was analyzed as PM5) that had lost RS1 and the TLC (toxin-linked cryptic) were obtained among 110 recombinants that were screened (Figure S3 and Table S2). After the removal of RS1 (PM4) and TLC:RS1 (PM5), the authentic *dif-*1 sequence (CCTTA**ATTTAACATAACATACATAATGCGCACT**AGGAACATTT) and the defective *dif*-like sequence (CCTTA**ATTTAACATAACATACATAATATGCACT**GAGGTATTTT) were restored, respectively, indicating that the excision of RS1 and TLC:RS1 was by a precise process. The exact mechanism of the excision of entire array remains unknown, although site-specific recombination through *att* sequences that flank the RS1s and CTX was proposed to explain the generation of Wave 2 strains [22]. The excision of RS1 and/or TLC:RS1 was also investigated in the *recA* ^−^ strain, and excision efficiencies were significantly reduced in the *recA* ^−^ strain, indicating that the excision of entire array from chromosome 1 might be mediated by an additional mechanism.

These results suggest that the entire array of chromosome 1 in V212-1 can be excised either by a stepwise manner, or by a single step process. A CTX-2 on chromosome 2 can also be excised, resulting in a strain that harbors a single CTX-2 on chromosome 2 (PM6).

The CTX arrays of some V212-1variants that we generated were identical to those in the clinical isolates of Wave 2 strains. PM2 has the TLC:RS1:RS1 array on chromosome 1 and CTX-2:CTX-2 on chromosome 2 and has the same structure as MG116926 [8]. PM5 has the same structure as B33 and MJ1236 [23]. Our results suggest that the intermediary strains such as V212-1 might be the immediate ancestor of Wave 2 strains.

### Infectious CTX-1 virions can be generated from chromosome 1 of V212-1

CTX virions can be generated from chromosome 1 of V212-1 and PM1, based on the replication mechanism of CTX phage [24]. *ctxAB* of CTX-1 of V212-1 and PM1 was replaced by a kanamycin cassette to construct strains PM7 and PM8, respectively, and the generation of kanamycin-resistant CTX virions from PM7 and PM8 was confirmed by generation of the kanamycin-resistant O395 transductant. The titer of CTX virions was approximately 10^4^ virions/ml and 10^5^ virions/ml in the culture supernatant of PM7 and PM8, respectively, in the presence of mitomycin C. pCTX-1kan generated from PM7 and pCTX-1-1kan generated from PM8 were extracted from the recipients and analyzed by sequencing. pCTX-1kan and pCTX-1-1kan had identical DNA sequences, because they were replication products from the same CTX-1 prophage genome of chromosome 1. No CTX-2 virions were generated from the tandem repeat of CTX-2 on chromosome 2 of PM9 and PM10, as no virions were produced from B33 [22].

### Generation of new mosaic CTX phages from V212-1

An inter-chromosomal recombination event between *ctxAB* ^El Tor^ of CTX-1 on chromosome 1 and *ctxAB* ^cla^ of CTX-2 on chromosome 2 in V212-1, generating new mosaic phage genomes, was verified by the generation of CTX-1*kan virions from strain PM9 (Table 2 and Figure 3). These CTX-1*kan virions were transduced into a recipient strain, from which pCTX-1*kan was obtained. The CTX-1*kan virion titer was 50 virions/ml in the presence of mitomycin C in PM9 culture supernatant (results of five independent experiments). The total number of CTX-1 virions and CTX-1*kan virions that were generated in PM9 should equal the number of CTX-1kan virions that were generated in PM7 (10^4^ virions/ml); thus, the efficiency of generation of CTX-1* virions from V212-1 was estimated to be 1 per 200 progeny virions. CTX-1* can be defined as a CTX phage that is identical to authentic CTX-1, except that it contains *ctxB* ^cla^.

The inter-strand recombination frequency was reduced to 1/10 in the PM9 *recA* ^−^ strain (6 virions/ml in culture supernatant of PM9 *recA* ^−^). This suggests that the inter-strand recombination is mainly mediated by homologous recombination; however, further detailed analysis is necessary to reveal the nature of the inter-strand recombination.

pCTX-1*kan was generated only from strain PM9, which has a kanamycin cassette in the first CTX-2 on chromosome 2. No CTX-1*kan was obtained from PM10, which contains the kanamycin cassette in the second CTX-2, in over 5 transduction experiments, possibly because the homologous region downstream of *ctxB* of the second CTX-2 is not long enough for recombination. However, under natural conditions without the kanamycin cassette, recombination between CTX-1 and the second CTX-2 should be possible. It needs to be elucidated if the inter-strand recombination event happens between two prophages (CTX-1 and CTX-2) or, between the replicative pCTX generated from chromosome 1 and the CTX-2 prophage. Perhaps, the recombination occurs more frequently between pCTX and the CTX-2 prophage than between two prophages.

PM11, PM12, and PM13 were generated similarly from strains that contained CTX-3^#^:RS1, CTX-5^#^:RS1, and CTX-6^#^:RS1, respectively, by replacing the *ctxAB* ^cla^ of CTX-2 with a kanamycin resistance cassette (Table 2). pCTX-3kan, pCTX-5kan, and pCTX-6kan were generated from PM11, PM12, and PM13, respectively, by inter-strand recombination like pCTX-1*kan (Table 2 and Figure 3).

These results demonstrate that new mosaic phages are generated through combination of inter-chromosomal recombination event between 2 phages and intra-strand recombination event between the resulting recombinant CTX phage genome and RS1 in an intermediary strain.

### Generation of Wave 3 strains

Because new mosaic CTX phages were generated from V212-1, we examined their transmission to novel El Tor strains to generate Wave 3 strains. Construction of the Wave 3 strains required a strain that contained only RS1 on chromosome 1, but no such strains were available among clinical isolates. PM14, which contains only RS1 on chromosome 1, was generated by the excision of CTX-1 from N16961. PM14 was transformed with various pCTXs that were extracted from transductants. pCTXs could be maintained in PM14 in plasmid form under selective antibiotic pressure or as an integrated prophage form. PM15, PM16, PM17, PM18, and PM19, which contained only the integrated pCTX-1kan, pCTX-1*kan, pCTX-3kan, pCTX-5kan, and pCTX-6kan next to the RS1 on chromosome 1, respectively, were screened in the transformants (Table 2).

These results demonstrate that the mosaic CTX phages that are produced from an intermediary strain such as V212-1 can be transmitted to an El Tor strain that contains an RS1 and can be integrated into the genome of a new host bacterial cell, enabling it to develop into a Wave 3 strain.

## Discussion

The emergence of atypical El Tor strains traces back to 1991 on the Indian subcontinent, where prototype and atypical El Tor strains began to co-existed for several years [6]. It is assumed that prototype El Tor strains have been extinguished, because only atypical El Tor strains have since been isolated from cholera patients globally [6]. Phylogenetic analyses that are based on SNPs in the *V. cholerae* genome have indicated that prototype and atypical El Tor strains originated from a common ancestor in the 1950's [10], [25]. Although the genome of El Tor *V. cholerae* strains has modulated gradually, the changes of their CTX phage have occurred stepwise manner. In addition, strains that harbor various CTX phages constitute phylogenetically distinct subgroups, implying that the acquisition of a new CTX phage is independent of evolution of the bacterial genome [10], [26].

Although several studies have proposed models for the generation of atypical El Tor strains, suggesting that the atypical variants originated through recombination events and lateral gene transfer, the exact process by which the mosaic CTX phages and atypical El Tor strains were generated remains unknown [16], [17], [22], [27]. In this study, we propose a genetic mechanism by which atypical El Tor variants are generated. We demonstrated how a single-segmented CTX virus reorganized its genome through recombination of 2 prophages and/or between a CTX prophage and RS1 in a host bacterial cell, resulting in a novel mosaic CTX virus, which can be transduced to a new host.

Homologous recombination between the common genes (*rstR* ^ET^, *rstA*, and *rstB*) between RS1 and CTX-1 or between any gene, except *rstR*, of the CTX-1 and CTX-2 (or, CTX^cla^), mediates generation of mosaic CTX phage genomes. Although the creation of CTX-2 was not shown directly—because no *V. cholerae* strains that have both CTX-1 and CTX^cla^ were available—our results support the existence of an ancestral strain that harbors CTX-1 and CTX^cla^ in nature. Further, *V. cholerae* O141 strains have been shown to be a reservoir of CTX^cla^, and an El Tor strain has been shown to take up CTX^cla^ from them [17].

Intra-strand recombination between CTX-1 and RS1 is essential not only for the generation of mosaic CTX phages but also for the emergence of Wave 3 *V. cholerae* strains that harbor these mosaic phages. CTX and/or RS1 can be excised from chromosome 1 if the array contains a CTX repeat or a CTX:RS1 repeat. When the array ends with RS1, the ultimate product of the excision is a strain that contains a lone RS1. Depending on the structure of the array, the excision rate can reach 1/(4.4×10^3^), indicating that a substantial proportion of the *V. cholerae* population tends to shed the CTX phage they harbor and is competent to acquire a new CTX phage. Thus, the Wave 3 atypical strains perhaps arose from prototype strains that had lost CTX-1 and obtained mosaic CTX phages that were generated from an intermediary strain.

**Figure 4:**
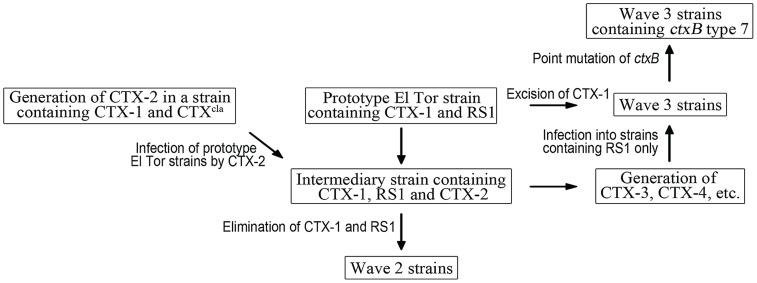
A model of the generation of Wave 2 and Wave 3 strains. Prototype El Tor strains transform into an intermediary strain by CTX-2 phage infection. It is unknown how the CTX-2 phage was generated, but we hypothesize that CTX-2 is generated in a strain containing CTX-1 and CTX^cla^. The intermediary stain is converted into Wave 2 strains by stepwise removal of CTX-1 and RS1. Mosaic CTX phages, such as CTX-3, CTX-4, are generated from the intermediary strain, and transduced into a new host bacterial strain that harbors only RS1 on chromosome 1, resulting in Wave 3 strains. *ctxB* type 1 of some Wave 3 strains is further converted into *ctxB* type 7 by a point mutation.

Based on these results, we propose a pathway by which atypical El Tor variants were generated from prototype El Tor strains (Figure 4). A prototype El Tor strain was infected by a CTX-2 virion, and the CTX-2 genome was integrated as a tandem repeat in the small chromosome—thus, the prototype strain turned into an intermediary strain. The prototype components were removed from the intermediary strain stepwise, and the resulting strains from each step developed into a group of Wave 2 strains. The intermediate strain was also a source of new mosaic phages of Wave 3 strains. New mosaic CTX phages were generated from the intermediary strain, and the progeny mosaic CTX phages were transduced to progenitor strains of Wave 3 strains.

We are interested in examining the dynamics of the emergence of Wave 2 and Wave 3 atypical strains. Classical biotype and prototype El Tor biotype strains coexisted for approximately 2 decades after the prototype El Tor strains first appeared in 1962 in South Asian countries [6]. Yet, inexplicably, the initial appearance of atypical strains was not documented until 1991—30 years after prototype El Tor strains originated. Mosaic CTX phages were most likely generated during the early seventh cholera pandemic period. Whether they were generated in the 1960s—when prototype El Tor strains and classical strains coexisted—and took 30 years to spread, or in the 1990s, shortly before dissemination into the human population, has not been determined. Further study is required to establish when the atypical strains were generated in their natural habitat and disseminated throughout the human population. Coincidentally, had the first atypical strains appeared around the early 1990s, their emergence would have concurred with that of the new O139 serogroup, which is also responsible for epidemic cholera.

The whole-genome analysis of the Haitian strains and a group of Nepalese strains showed that their genomes differ by 1 or 2 base pairs, indicating that the Haitian *V. cholerae* strains originated from Nepal [28]. The strain that was isolated from the 2010 Haiti cholera outbreak contained CTX-6b and the proportion of strains that contain *ctxB* genotype 7 has been increasing rapidly in Kolkata, India, since it first emerged in 2006, [13]. Strains that harbored CTX-6b had not been found until 2007 in India, whereas a strain with CTX-6 was isolated in 2007. We hypothesize that mosaic CTX phages that contain *ctxB* genotype 7 emerged through 2 possible mechanisms: *ctxB* of the intermediary strain changed first, and progeny mosaic viruses that harbored *ctxB* genotype 7 were produced; alternatively, CTX-3b and CTX-6b independently arose from CTX-3 and CTX-6 by point mutation of *ctxB*. CTX-3b and CTX-6b are the CTXs that harbor *ctxB* genotype 7 that have been identified, but the existence of CTX-4b or CTX-5b or new types of CTXs should be examined. A surveillance study of the generation and dissemination of strains that contain *ctxB* genotype 7 will provide evidence on how the *V. cholerae* population has changed and aid in tracking strains with specific CTX prophage.

A link between the increase in severe cholera cases and atypical *V. cholerae* strains has been proposed, but the precise mechanism by which the clinical symptoms and genetic changes in the bacterial strains correlate remains unknown [29]. Although our results demonstrate the mechanism of the genesis of atypical strains, further studies are required to explain how new variants differ in pathology and how they dominate earlier prevalent strains. It is unclear whether population changes in clinically isolated *V. cholerae* strains reflect those of *V. cholerae* in the natural habitat. In addition to bacterial and environmental factors, human activity and, perhaps, the human immune response against temporarily prevalent strains influence population changes in clinically isolated *V. cholerae* strains.

## Materials and Methods

### Bacterial strains and genetic structure analysis

The bacterial strains and pCTX are shown in Tables 1 and 2. The CTX and RS1 arrays of the *V. cholerae* strains were determined and analyzed by sequencing. The DNA sequences of the CTX arrays of bacterial strains and pCTXs that were generated in this study were deposited into GenBank (Table 2).

### Discrimination PCR of SNPs of *rstB*

A PCR primer set was designed for detection of the trinucleotide GTA (nt position 74–76) in *rstB*, similar to MAMA PCR for distinguishing classical, El Tor, and Haitian type *ctxB* [13]. For the Wave 3 strains that contained the RS1:CTX array, the forward primer, rstBFw (CTC ATT CTG AAG GGG TGA GTA A) was designed to anneal to *rstB* of RS1 (Figure S1), and the reverse primer, GTARev (GGT GCA CCA GTC TTA CAA C) was designed to detect GTA with rstBFw (annealing temperature at 63°C). The primer DelRev (GCA CCA GTC TTA CGT AC) was designed to verify the absence of GTA with rstBFw (annealing temperature 58°C).

### Surveillance of various CTX prophage genomes in Wave 3 strains

The GTA trinucleotide was examined in *rstB* in 365 clinical isolates of Wave 3 strains that were collected from 2004 to 2007 in Kolkata, India and 20 isolates that were gathered in Haiti in 2010 [14], [19]. DMAMA (Double-Mismatch-Amplification Mutation Assay)-PCR for *ctxB* was also performed with these isolates. CTX phages that contained GTA-deleted *rstB* were confirmed by DNA sequencing of the entire RS1:CTX array.

### Excision of the CTX and RS1 from chromosome 1 and chromosome 2 of V212-1

A DNA fragment (690 bp) that contained *rstR* ^ET^ (339 bp) and the first 226 bp of *rstA* was inserted into pCVD442 to generate a recombinant plasmid, pCVDrstRET. The recombinant plasmid was inserted into *rstR* ^ET^ of CTX-1 and RS1s of V212-1 to construct V212-1CVD [30]. The excision of CTX-1and/or RS1 was verified by analyzing the genetic structure of strains that were selected on LB plates with 15% sucrose. In addition, pCVDrstRET was inserted into *rstR* ^ET^ of PM3 to construct PM3CVD, and excision of the RS1 element was confirmed as described. Excision of CTX-2 from the tandem repeat of CTX-2 on chromosome 2 was examined similarly by inserting *rstR* ^cla^ fragment-containing pCVD442 into each *rstR* ^cla^ of CTX-2s.

### Replacement of *ctxAB* in V212 with a kanamycin cassette

The entire *ctxA* gene and the first 166 bp (of 375 bp) of *ctxB* of CTX-1 on chromosome 1 and CTX-2s on chromosome 2 of V212-1 were individually replaced by a kanamycin resistance cassette by allele exchange method [21]. The residual *ctxB* fragment on each recombinant strain contained the second SNP (nucleotide 203) of *ctxB* ^El Tor^ (T) and *ctxB* ^cla^ (C) on chromosomes 1 and 2, respectively. This SNP was used to distinguish pCTX-1kan and pCTX-1*kan. Similarly, a kanamycin cassette was introduced into CTX-1 on chromosome 1 of PM1, resulting in strain PM8. Strains PM11, PM12, and PM13 were constructed similarly by replacing the *ctxAB* ^cla^ of the first CTX-2 on chromosome 2 with a kanamycin cassette.

### Production of CTX-1 phage from chromosome 1 of V212-1

Transduction of kanamycin-resistant CTX virions was monitored as described [21], [31]. Briefly, PM7 culture supernatant (LB containing 20 ng/ml of mitomycin C) was mixed with a classical strain, O395, as the recipient. The mixture was incubated for 30 min for phage infection and plated on LB plates that contained kanamycin. pCTX-1kan, a replicative form of the CTX genome in the transductants, was extracted and analyzed by sequencing. CTX-1-1kan virions were generated similarly from PM8 and were determined to have the same DNA sequence as CTX-1kan (Table 2).

### Generation of Wave 3 strains

PM14 was generated from N16961 by excision of CTX-1. The recombinant plasmid pCVDrstRET was inserted into CTX-1 of N16961, and strain PM14 was screened as described above. PM14 was transformed by the replicative form of CTXs that were generated in this study (pCTX-1kan, pCTX-1*kan, pCTX-3kan, pCTX5kan, and pCTX-6kan). Integration of pCTXs next to RS1 on chromosome 1 of PM14 was confirmed by DNA sequencing (Table 1) [8].

### *recA* ^−^ mutants construction

The internal 600 nt DNA fragment (nucleotide position 119–720) of *recA* was amplified with primers recASacIF: CGC GCG GAG CTC CGA CCC GAT TTC GAC ACA and recAEcoRIR: CCG GGC GAA TTC CTT GAG GTT ACC CGT CAG by using PCR. This fragment was inserted into a suicide plasmid pSW23.oriT to construct a recombinant plasmid pSWrecA [32]. The recombinant plasmid was individually transferred by conjugation to V212-1CVD, PM3CVD, and PM9. The *recA* of each strain was disrupted by insertion of pSWrecA.

### Accession numbers

The nucleotide sequences of CTX prophages and pCTXs were deposited in GenBank under accession numbers KF471410, KF664566–KF664576, KF664579, KF664580, and KJ540257–KJ540278.

## Supporting Information

Figure S1 **Discriminatory PCR of** ***rstB*** **in CTX-3 and CTX of Haitian strain.** (A) *rstB*-discriminating PCR primers. The common *rstB* forward primer is shown on top. DNA sequences of *rstB* of CTX-3 (nucleotides 65–88, shown in bold) and Haitian CTX aligned with reverse primers. Three nucleotides, GTA (nt position 74–76, underlined), are absent in *rstB* of CTX of the Haitian strain. (B) Agarose gel electrophoresis of PCR product using rstBFw/GTARev (left panel) and rstBFw/DelRev (right panel). Lane 1: IB4122, lane 2: Haiti strain, lane 3: IB4247, lane 4: IB4712. (C) Primer annealing location on RS1-CTX array of Wave 3 strains. The size of amplicon is approximately the same as an RS1 element.
(TIF)

Figure S2 **Excision of CTX-1 and RS1 from chromosome 1 of V212-1.** (A) RS1:CTX-1:RS1 array on chromosome 1 of V212-1. (B) pCVDrstRET (red bar) was inserted into the *rstR* of CTX-1 of V212-1, generating a recombinant strain, V212-1CVD. Excision of the recombinant plasmid was screened by inoculating the strain V212-1CVD on LB agar plates containing 15% sucrose. Potential recombination positions are indicated (the dotted line also shows a potential recombination position that is not detected during the screening). (C) The CTX and RS1 arrays generated from each recombination shown in B. Generation of RS1:CTX-1:RS1 and RS1:RS1 is mediated by recombination between *rstR*, but the generation of CTX-1:RS1 and RS1 arrays occurs through recombination between the entire RS2 region (*rstR*, *rstA*, and *rstB*) of RS1 and CTX-1. TLC is not shown in this figure. The overall excision rate and frequencies of generation of each array are described in Table 3.
(TIF)

Figure S3 **Generation of PM4 and PM5 from PM3.** pCVDrstRET (red bar) was inserted into the *rstR* of RS1 in PM3 to construct PM3CVD, and the recombinant strain was inoculated on LB plates containing 15% sucrose to screen for excision of pCVDrstRET. Most strains (105 strains) had the same array as PM3, but one strain (PM4) that had lost the RS1, and 4 strains that had lost RS1 and TLC were obtained (PM5).
(TIF)

Table S1 **Distribution of CTX phages in Wave 3 strains collected in India between 2003 and 2007.**
(DOCX)

Table S2 **Frequency of generation of each array from PM3CVD and PM3CVD** ***recA^−^*** **.** Each array can be generated by recombination events shown in Figure S3.
(DOCX)
