# Supplementary figures and images for: Molecular Insights Into the Evolutionary Pathway of Vibrio cholerae O1 Atypical El Tor Variants
Source: PLoS Pathog. 2014 Sep 18;10(9):e1004384. doi: 10.1371/journal.ppat.1004384 (PMC4169478; doi:10.1371/journal.ppat.1004384)

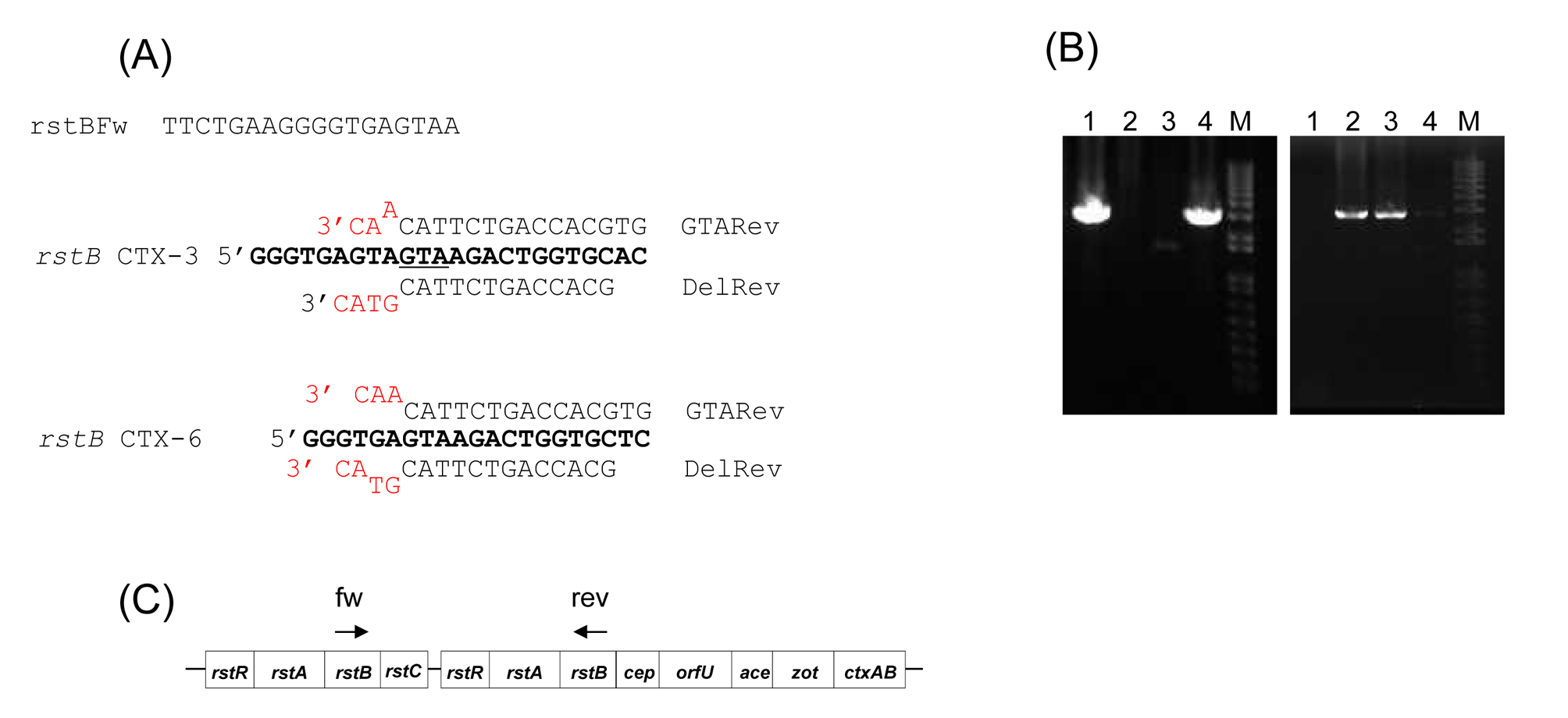

Supplement: Figure S1 — Discriminatory PCR of rstB in CTX-3 and CTX of Haitian strain. (A) rstB-discriminating PCR primers. The common rstB forward primer is shown on top. DNA sequences of rstB of CTX-3 (nucleotides 65–88, shown in bold) and Haitian CTX aligned with reverse primers. Three nucleotides, GTA (nt position 74–76, underlined), are absent in rstB of CTX of the Haitian strain. (B) Agarose gel electrophoresis of PCR product using rstBFw/GTARev (left panel) and rstBFw/DelRev (right panel). Lane 1: IB4122, lane 2: Haiti strain, lane 3: IB4247, lane 4: IB4712. (C) Primer annealing location on RS1-CTX array of Wave 3 strains. The size of amplicon is approximately the same as an RS1 element. (TIF) [file ppat.1004384.s001.tif]

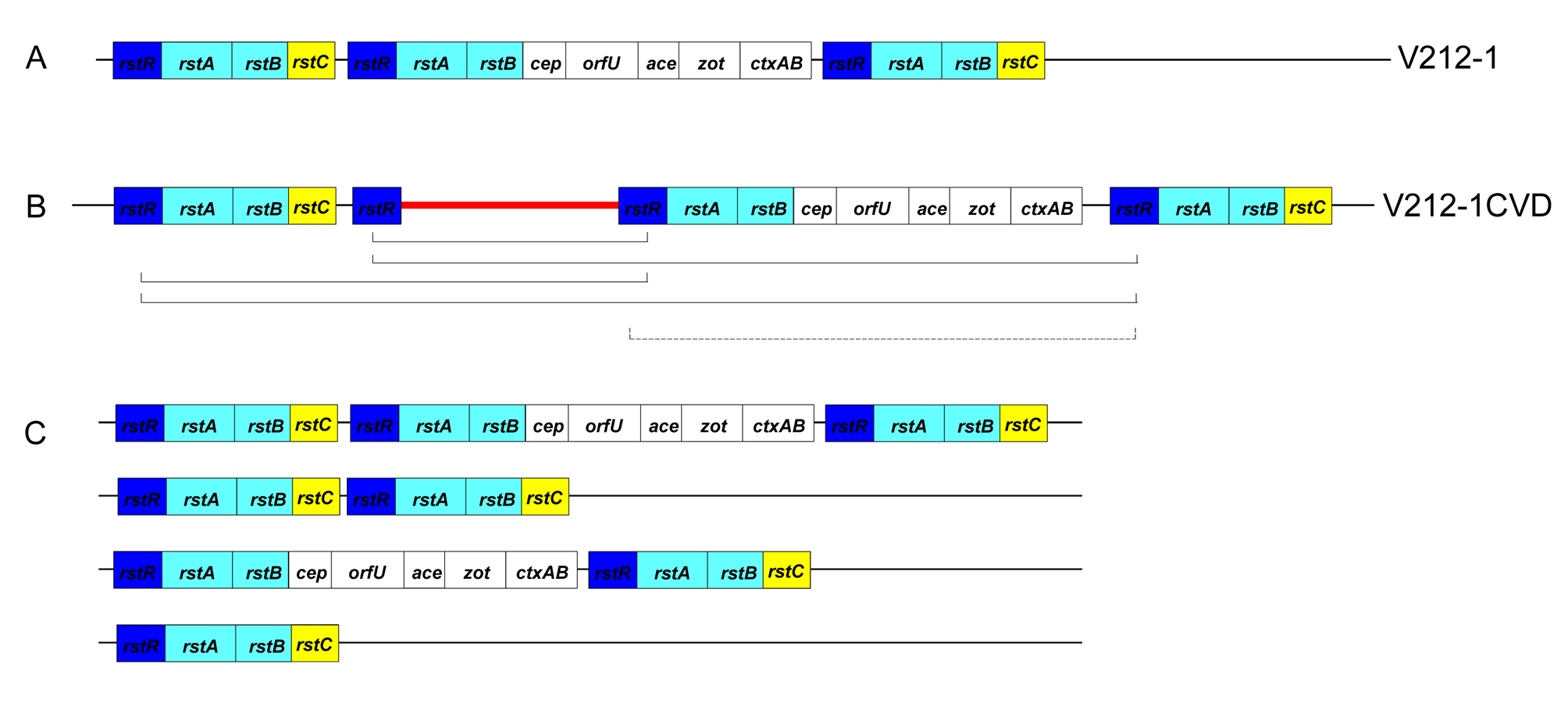

Supplement: Figure S2 — Excision of CTX-1 and RS1 from chromosome 1 of V212-1. (A) RS1:CTX-1:RS1 array on chromosome 1 of V212-1. (B) pCVDrstRET (red bar) was inserted into the rstR of CTX-1 of V212-1, generating a recombinant strain, V212-1CVD. Excision of the recombinant plasmid was screened by inoculating the strain V212-1CVD on LB agar plates containing 15% sucrose. Potential recombination positions are indicated (the dotted line also shows a potential recombination position that is not detected during the screening). (C) The CTX and RS1 arrays generated from each recombination shown in B. Generation of RS1:CTX-1:RS1 and RS1:RS1 is mediated by recombination between rstR, but the generation of CTX-1:RS1 and RS1 arrays occurs through recombination between the entire RS2 region (rstR, rstA, and rstB) of RS1 and CTX-1. TLC is not shown in this figure. The overall excision rate and frequencies of generation of each array are described in Table 3. (TIF) [file ppat.1004384.s002.tif]

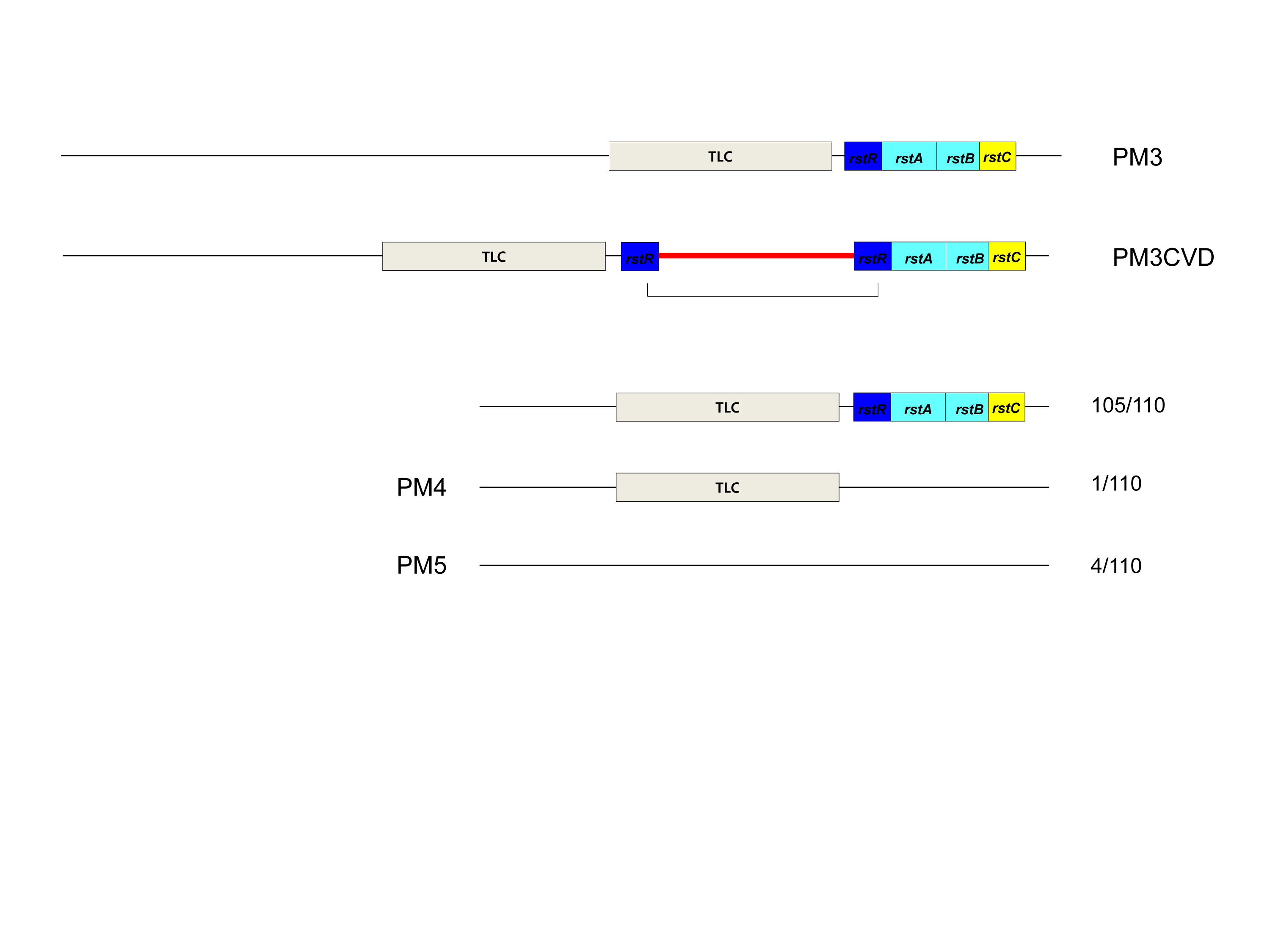

Supplement: Figure S3 — Generation of PM4 and PM5 from PM3. pCVDrstRET (red bar) was inserted into the rstR of RS1 in PM3 to construct PM3CVD, and the recombinant strain was inoculated on LB plates containing 15% sucrose to screen for excision of pCVDrstRET. Most strains (105 strains) had the same array as PM3, but one strain (PM4) that had lost the RS1, and 4 strains that had lost RS1 and TLC were obtained (PM5). (TIF) [file ppat.1004384.s003.tif]
