# Supplementary material for: Molecular Insights Into the Evolutionary Pathway of Vibrio cholerae O1 Atypical El Tor Variants
Source: PLoS Pathog. 2014 Sep 18;10(9):e1004384. doi: 10.1371/journal.ppat.1004384 (PMC4169478; doi:10.1371/journal.ppat.1004384)
Supplement: Table S1 — Distribution of CTX phages in Wave 3 strains collected in India between 2003 and 2007. (DOCX) [file ppat.1004384.s004.docx]

**Table S1. Distribution of CTX phages in Wave 3 strains collected in India between 2003 and 2007.**

| Year | CTX-3 | CTX-3b | CTX-4 | CTX-5 | CTX-6 | Total |
| --- | --- | --- | --- | --- | --- | --- |
| 2003 | 53 | 0 | 0 | 1 | 0 | 54 |
| 2004 | 85 | 0 | 0 | 1 | 0 | 86 |
| 2005 | 27 | 0 | 0 | 7 | 0 | 34 |
| 2006 | 37 | 68 | 0 | 0 | 0 | 105 |
| 2007 | 70 | 14 | 1 | 0 | 1 | 86 |
| Total | 272 | 82 | 1 | 9 | 1 | 365 |
