# Supplementary material for: Molecular Insights Into the Evolutionary Pathway of Vibrio cholerae O1 Atypical El Tor Variants
Source: PLoS Pathog. 2014 Sep 18;10(9):e1004384. doi: 10.1371/journal.ppat.1004384 (PMC4169478; doi:10.1371/journal.ppat.1004384)
Supplement: Table S2 — Frequency of generation of each array from PM3CVD and PM3CVD recA− . Each array can be generated by recombination events shown in Figure S3. (DOCX) [file ppat.1004384.s005.docx]

|  | TLC:RS1 | TLC | No element | Recombination efficiency |
| --- | --- | --- | --- | --- |
| PM3CVD,  2 × 10^6^ cells Inoculated | 21 | 0 | 0 | 22/2×10^6^ cells = 1/(9.1×10^4^) |
|  | 18 | 1 | 1 |  |
|  | 24 | 0 | 0 |  |
|  | 19 | 0 | 3 |  |
|  | 23 | 0 | 0 |  |
| PM3CVD *recA^-^*,  2 × 10^7^ cells Inoculated | 2 | 0 | 0 | 2.4/2×10^7^ cells = 1/(8.3×10^6^) |
|  | 2 | 0 | 0 |  |
|  | 1 | 0 | 0 |  |
|  | 3 | 0 | 0 |  |
|  | 3 | 0 | 1 |  |

**Table S2. Frequency of generation of each array from PM3CVD and PM3CVD *recA^-^***. Each array can be generated by recombination events shown in Figure S3.

Results of five independent experiments are shown.
